# Myospheres Are Composed of Two Cell Types: One That Is Myogenic and a Second That Is Mesenchymal

**DOI:** 10.1371/journal.pone.0116956

**Published:** 2015-02-23

**Authors:** Karen A. Westerman

**Affiliations:** Department of Anesthesia, Perioperative and Pain Medicine, Brigham and Women’s Hospital, Boston, Massachusetts, United States of America; University of Minnesota Medical School, UNITED STATES

## Abstract

Previously, in an attempt to isolate stem cells that would be capable of regenerating injured skeletal muscle, we cultured cells derived from muscle, non-adherently, in serum-free media. As a result of the culture conditions used, these cells formed spheres, and thus were referred to as myospheres. It was found that myosphere-derived cells expressed Sca-1, a marker that is not typically associated with myogenic cells, and as a result has generated some questions as to the origin of these cells. The goal of this study was to clearly determine the origin of myosphere-derived cells, and in particular to answer the question of whether myospheres contain myogenic cells. To determine if myospheres were composed of myogenic cells without altering the structure of myospheres or the culture conditions used to maintain myospheres, I isolated these cells from yellow fluorescent protein (YFP)-Myf5, YFP-MyoD, and ZsGreen-Pax7 lineage-tracing mice and monitored their growth over time. I found that myospheres do contain myogenic cells, but that these cells are gradually lost over time (within 2 months). Additionally, the use of the lineage-tracing mice gave an interesting perspective into the composition of myospheres. I found that myospheres were composed of two distinct cell types, one that is myogenic (α7 integrin^+^) and contains cells expressing Myf5, MyoD, and Pax7, and a second that is non-myogenic (α7 integrin^-^) expressing platelet-derived growth factor receptor alpha (PDGFRα) and Sca-1, both of which have been associated with fibro/adipocyte mesenchymal cells.

## Introduction

One of the greatest challenges to using cell-based therapies to treat muscle disease is the ability to isolate, expand, and deliver suitable donor cells needed for transplantation. This challenge is further complicated by the fact that diseased muscle is constantly repairing itself, going through periods of degradation and regeneration, indicating that in order to achieve a long-term engraftment, the donor cells chosen should have the potential of contributing to the existing muscle stem cell population, referred to as satellite cells. Satellite cells are mononuclear cells that sit adjacent to the myofibers but just beneath the basal lamina [1]. These cells give rise to myoblasts [2,3], which have been shown to repair injured muscle by fusing with the existing myofibers [4,5,6,7,8]. Satellite cells are recognized by their expression of transcription factor Pax7 [9], the loss of which has been linked to changes in satellite cell proliferation and differentiation [10,11,12]. Additionally, satellite cells have the ability to self-renew, further establishing their potential as muscle stem cells [13,14,15]. While these studies and many others have established satellite cells play an important role in the maintenance and repair of skeletal muscle, it was only recently shown that satellite cells are absolutely required for the regeneration of injured muscle, this was clearly demonstrated by the complete loss of muscle regeneration after selective ablation of the satellite cell population in adult mice [16,17,18]. Taken together, these factors all indicate that satellite cells will make the best donor cell candidate to achieve a successful cell engraftment. Unfortunately, attempts made to use expanded satellite cell pools, as donor cells, have not been successful because when these cells are expanded in culture they mature and lose their ability to engraft [19,20]. Alternatively, the use of freshly isolated satellite cells have shown great promise for cell transplantation, however the small number of cells that can be obtained and the need for immediate transplantation limits their potential as donor cells in a clinical situation [14,21,22]. In an attempt to find a suitable stem cell source that could be used to regenerate skeletal muscle, my lab examined an alternative method of isolating muscle-derived cells. This method involved culturing muscle-derived cells non-adherently as spheres in serum-free media; the resulting cell structures were referred to as myospheres [23]. The initial rational behind this unconventional culturing method was that the 3-dimensional cell-cell interactions would provide a niche-like environment to help maintain cells in a more primitive state [24]. One of the advantages of culturing myospheres is that they can be easily isolated from both young and old mice and they can be cultured long periods of time (3–4 months). The initial characterization of myosphere cultures indicated that these cells were interstitial cells because they expressed Sca-1 [23,25,26], and because they did not appear to express myogenic markers (MyoD or Pax7) [23]. However, we also found that cells derived from myospheres could express MyoD and Pax7 as well as form multinucleated myotubes when cultured adherently in the appropriate culture media, and that cells that had remained in culture as myospheres for 1 month were able to engraft into injured muscle fibers [23]. Combined, these data indicated that at some point myospheres must contain myogenic cells. Here I report that myospheres are composed of two cell populations, one that is clearly myogenic, expressing MyoD and α7 integrin, and a second population expressing platelet-derived growth factor receptor alpha (PDGFRα), which has been shown to be associated with the fibro/adipocyte mesenchymal cells [27,28,29]. Additionally and most importantly, I found that within the population of myogenic myosphere-derived cells, there is a subpopulation of cells that express Pax7.

## Materials and Methods

### Animals

Animals used in this study include: 25 wild type C57BL/6 mice (4–12 weeks old, obtained from Charles River Laboratories; Wilmington, MA, USA), 2 yellow fluorescent protein (YFP)-Myf5 and 5 YFP-MyoD mice (4–10 months old, obtained by crossing B6.129X1-Gt(ROSA)26Sor mice with Myf5^*Cre*^ (both from Jackson Laboratory; Bar Harbor, ME, USA) or MyoD^*iCre*^ [30] mice), and 14 ZsGreen Pax7 (1 ½- 5 months old with a C57BL/6 background, obtained from Dr. Michael Kyba [31]). Animal protocols used in this study were approved by the Harvard Medical Area Standing Committee on Animals (Assurance number A3431–01).

### Isolation and Culturing of Myospheres

Myospheres were isolated and maintained as we previously described in [23]. Briefly, hind limb muscles were isolated from mice and then enzymatically dissociated by a mixture of 2.4 U/ml dispase and 10 mg/ml collagenase A (both from Roche; Indianapolis, IN, USA) for 1 hour at 37°C. After digestion the slurry was dissociated further using a scalpel and then F10 media (Life Technologies; Grand Island, NY, USA) containing 20% FCS (fetal calf serum) (Hyclone; Logan, UT, USA) was added to inactivate the dispase/collagenase. The slurry was passed through a 70 μm cell strainer (BD Falcon; Franklin Lakes, NJ, USA) and centrifuged for 15 minutes at 156 x g. Cell pellets were resuspended in 1 ml of red blood cell lysis buffer (0.15 M ammonium chloride/ 0.01 M potassium bicarbonate solution, pH 7.4) for 2½ minutes on ice. After lysis 20 ml of 1:1 DMEM (Dulbecco’s modified Eagle’s medium):F12 was added and the cells were pelleted and then resuspended in 5 ml of B27 media (1:1 DMEM:F12 media containing B27supplement and 100 U/ml penicillin-streptomycin (all from Life Technologies)), triturated, filtered through 40 μm cell strainer (BD Falcon; Franklin Lakes, NJ, USA), and then brought up and cultured in B27 media supplemented with 20 ng/ml bFGF (basic fibroblast growth factor), 20 ng/ml hEGF (human epidermal growth factor) (both from PeproTech; Rocky Hill, NJ, USA), and 2 μg/ml heparin (Stem Cell Technologies; Vancouver, BC, Canada). Myospheres are maintained by feeding growth factors 2X a week and by adding additional B27 media as needed due to evaporation. Myospheres >100 μm are passaged by dissociating the spheres into single cell suspension using dispase/collagenase, cells are washed 1X with 1:1 DMEM:F12, and then plated in fresh B27 media with growth factors at a density of 2.5–3 X 10^5^ cells/ml (every 10–14 days).

### Cell Counting of Myosphere-Derived Cells

Fluorescence microscopy was used to monitor single cell suspensions of myosphere-derived cells isolated from YFP-Myf5^Cre^, YFP-MyoD^iCre^, and ZsGreen-Pax7 mice. Cells isolated from YFP-Myf5^Cre^ and YFP-MyoD^iCre^ were monitored for the expression YFP, which indicated that those cells either express or expressed Myf5 or MyoD, respectively. Cells isolated from ZsGreen-Pax7 mice were monitored for the expression of ZsGreen, indicating that those cells were currently expressing Pax7. Cell counts were taken immediately after isolation, during the first 6 days after isolation (before the formation of spheres), and at intermittent time points when the spheres were dissociated into single cell suspensions for passaging. Between 3–10 fields were counted for each time point analyzed. Myosphere-derived cells isolated from wild type mice were used as controls. Myosphere-derived cells were monitored using an Olympus IX70 fluorescence microscope (Olympus; Center Valley, PA, USA) equipped with a 20X lens along with YFP and GFP filters. Pictures were taken with a digital camera using Advantage Software version 4.5 (Spot Imaging Solutions, Sterling Heights, MI).

### Myogenic Differentiation

Myospheres isolated from YFP-MyoD mice were dissociated using a mixture of 2.4 U/ml dispase and 10 mg/ml collagenase A, sorted for the myogenic (YFP^+^) and mesenchymal (YFP^-^) populations, and then cultured adherently on collagen-coated plates in myoblast media (F10 media containing 20% FCS, 100 U/ml penicillin-streptomycin, and 5 ng/ml bFGF) as described previously [23]. To promote myotube differentiation, adherently cultured myosphere-derived cells were plated in F10 media containing 20% FCS and 100 U/ml penicillin-streptomycin in 24 well dishes coated with ECL (entactin-collagen IV-laminin cell attachment matrix) (Upstate/Millipore; Billerica, MA, USA). Media was changed the following day to DMEM media containing 5% heat inactivated horse serum and 100 U/ml penicillin-streptomycin, 3–5 days after media change myotubes were fixed with ice-cold methanol for 20 minutes and then the nuclei were stained using DAPI (4”, 6-diamidino-2-phenylindole dihydrochloride) (Sigma; St. Louis, MO, USA) for 5 minutes at 20°C. Myotube pictures were taken using a BZ-9000 fluorescence microscope (Keyence; Elmwood, NJ, USA) with BZ-II viewer software.

### CellTrace Violet Cell Proliferation Assay

Single cell suspensions of myosphere-derived cells (5–6 X10^6^ cells) were incubated at 37°C for 20 minutes with 10 μl of 5 mM CellTrace Violet (Life Technologies) diluted in 2 ml of HBSS (Hank’s balanced salt solution). Excess CellTrace was removed by incubating the cells with 10 ml of B27 media for 5 minutes at 37°C, after which cells were centrifuged for 10 minutes at 156 x g and then resuspended in fresh B27 media containing 20 ng/ml bFGF, 20 ng/ml hEGF, and 2 μg/ml heparin. CellTrace labeled myosphere-derived cells were monitored by an Olympus IX70 fluorescence microscope, (Olympus; Center Valley, PA, USA) using a standard DAPI filter, and by flow cytometry using the Pacific blue channel. Pictures were taken with a digital camera using Advantage Software version 4.5 (Spot Imaging Solutions, Sterling Heights, MI).

### Flow Cytometry

All analysis and cell sorting was done by a BD FACSAria (BD Biosciences; Franklin Lakes, NJ, USA) at the Joslin Diabetes FACS core facility. Prior to analysis or sorting, myospheres were dissociated into a single cell suspension using a mixture of 2.4 U/ml dispase and 10mg/ml collagenase A and then washed 1X with DMEM:F12 and 1X with HBSS containing 0.1% BSA (bovine serum albumin) (both from Life Technologies). Cells were incubated with conjugated antibodies for 45 minutes on ice. After antibody incubation, myosphere-derived cells were washed 2X in HBSS containing 0.1% BSA and then resuspended in 300 μl HBSS containing 0.5% BSA for analysis. Cells were labeled using APC (allophycocyanin) conjugated antibodies to α7 integrin (R&D Systems; Minneapolis, MN, USA), PDGFRα (eBioscience; San Diego, CA, USA), or Sca-1 (BioLegend; San Diego, CA, USA). Some cell populations were also labeled with CellTrace Violet. Cell sorting included myosphere-derived cells isolated from YFP-MyoD^Cre^ mice, which were sorted for the YFP^+^ and YFP^-^ cell populations. Myosphere-derived cells isolated from *wt* mice that were labeled with Sca-1-FITC (fluorescein isothiocyanate) (BD Phamingen, San Diego, CA, USA), were used as a control and to set up the YFP gate. Additionally, myosphere-derived cells isolated from *wt* mice were labeled with CellTrace and then incubated with either α7 integrin or PDGFRα antibodies. These cells were then sorted for cells expressing the CellTrace dye in combination with the corresponding antibody-positive and-negative cell populations (for α7 integrin and PDGFRα). Myosphere-derived cells isolated from *wt* mice were labeled with either the CellTrace dye or APC (α7 integrin or PDGFRα antibodies) and then used as controls to set the pacific blue and APC gates. Propidium iodide (Life Technologies) was added to all sorts to gate out dead cells. For each sample 10,000–100,000 cells were analyzed. Unstained cells were used as a negative control. Data analysis was done using FlowJo (TreeStar, Inc; Ashland, OR, USA).

### Statistical analysis

In all experiments, results are expressed as means ± s.e.m. Statistical differences between two sets of data were determined using the unpaired Student’s t test; p < 0.05 was considered statistically different. All statistical calculations were performed using GraphPad Prism 6 software (GraphPad Software; La Jolla, CA, USA).

## Results

### Myospheres contain myogenic cells

**Fig 1:**
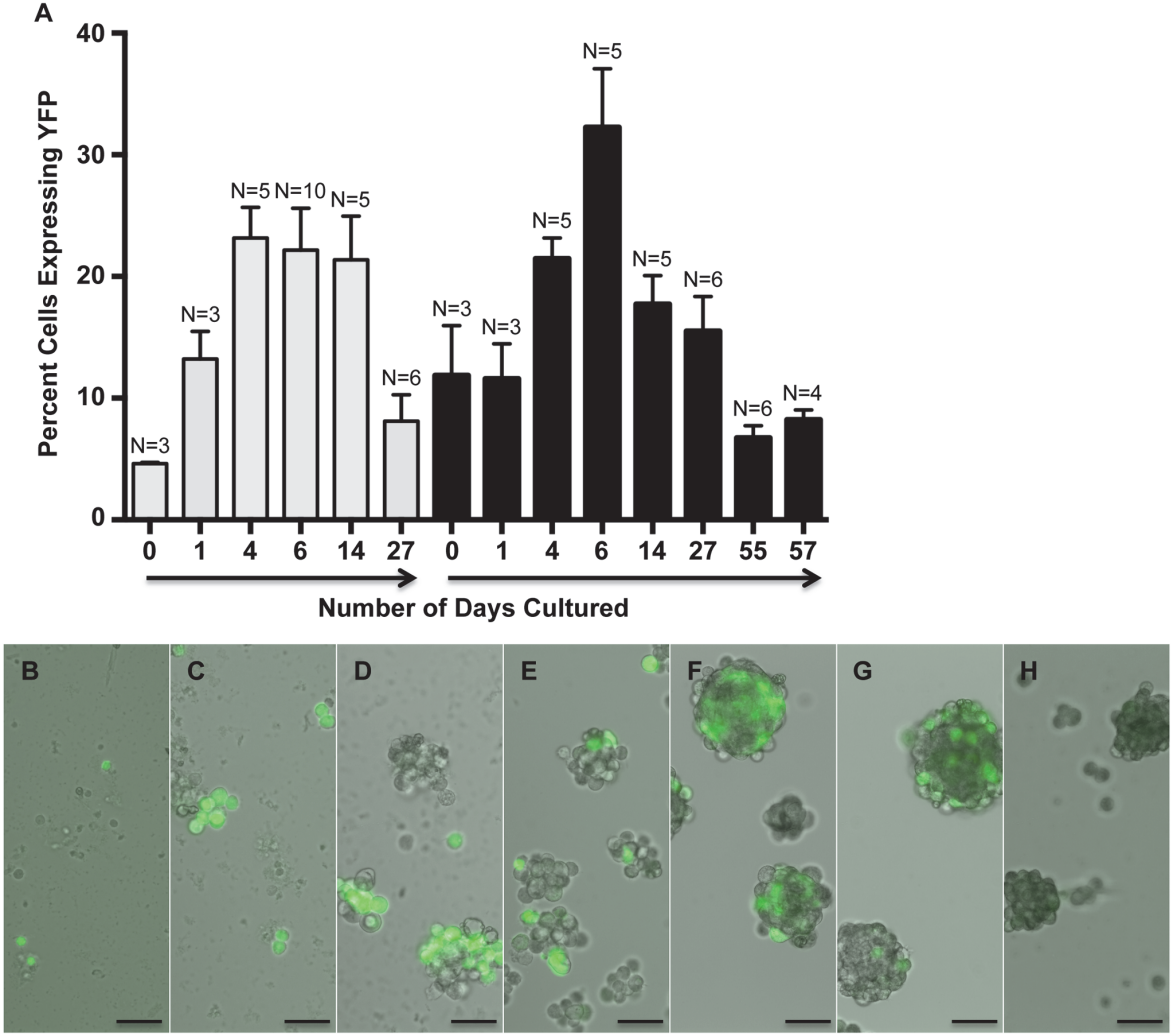
Myosphere-derived cells express Myf5. (A) Graph showing the percentage of myosphere-derived cells isolated from YFP-Myf5 mice that express YFP versus the number of days these cells had remained in culture. Shown are two independent myosphere isolations. N refers to the number of random fields counted. (B-H) Representative pictures showing the growth of myospheres and the expression of YFP in myosphere cultures isolated from YFP-Myf5 mice. Shown are pictures from (B) 1, (C) 4, (D) 6, (E) 15, (F) 22, (G) 26, and (H) 32 days after isolation. Scale bar represents 50μm.

**Fig 2:**
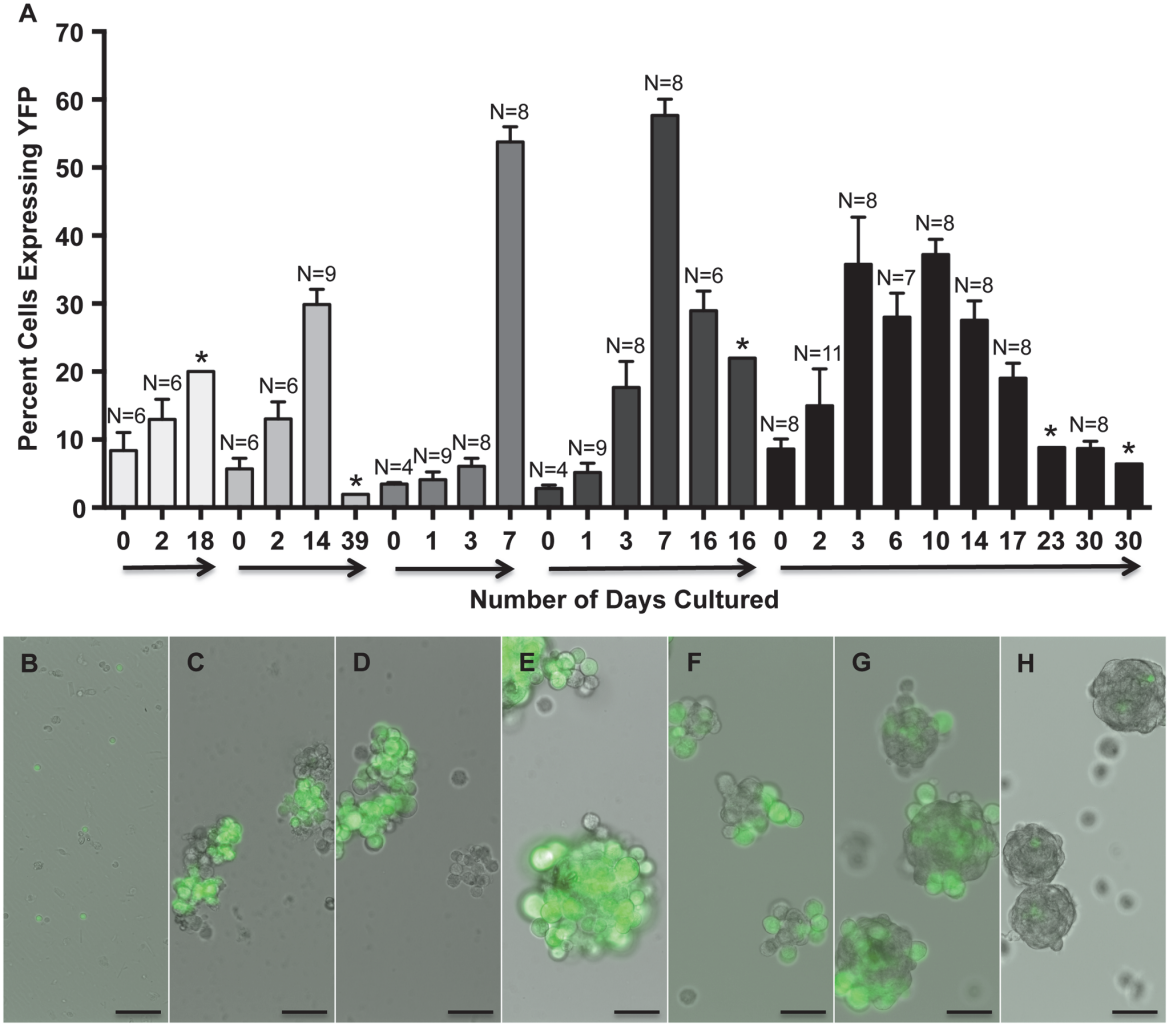
Myosphere-derived cells express MyoD. (A) Graph showing the percentage of myosphere-derived cells isolated from YFP-MyoD mice that express YFP versus the number of days these cells had remained in culture. Shown are five independent myosphere isolations. N refers to the number of random fields counted. * Refers percentages obtained by FACS. (B-H) Representative pictures showing the growth of myospheres and the expression of YFP in myosphere cultures isolated from YFP-MyoD mice. Shown are pictures from (B) 0, (C) 4, (D) 6, (E) 10, (F) 16, (G) 20, and (H) 28 days after isolation. Scale bar represents 50μm.

**Fig 3:**
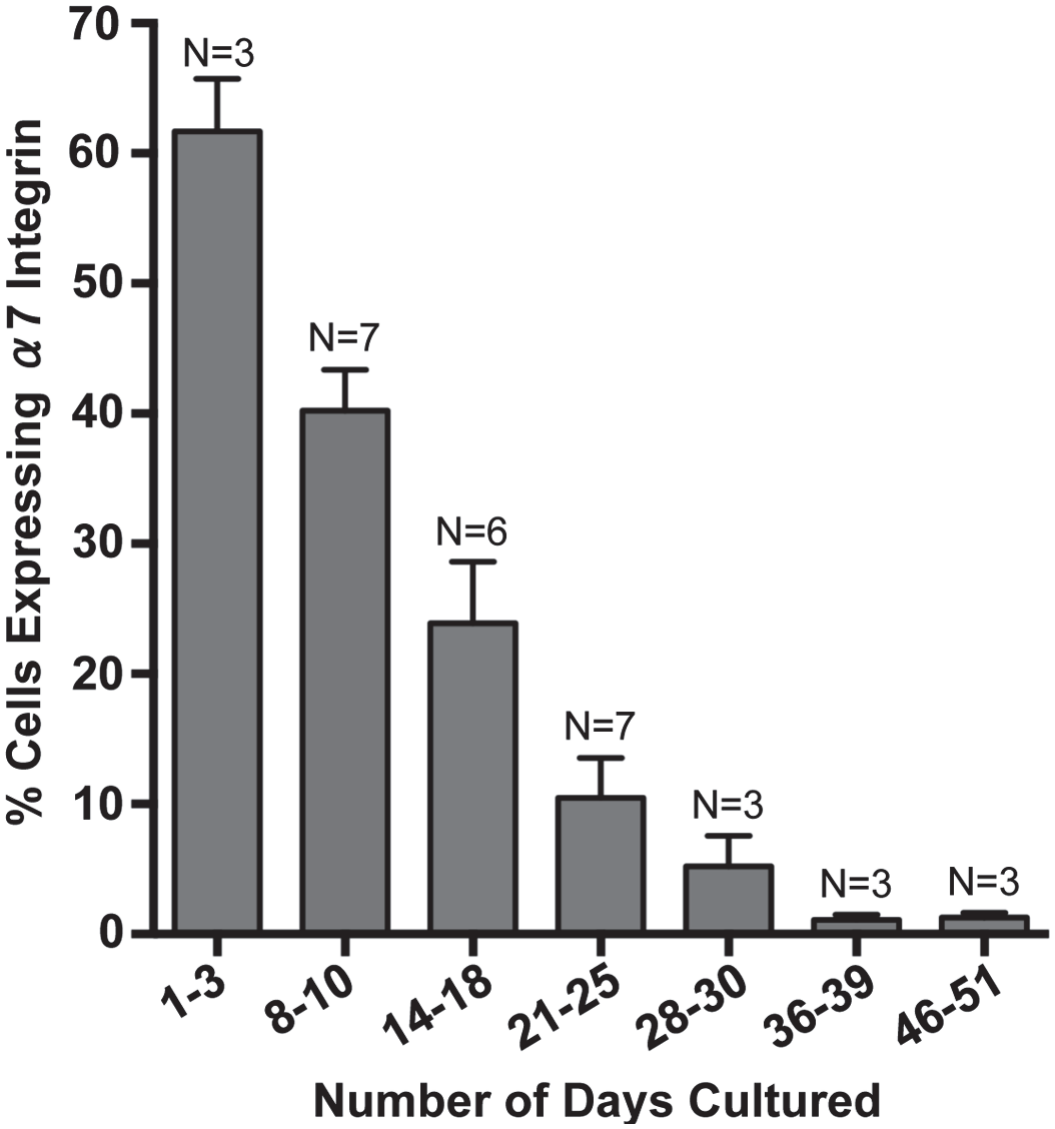
Myosphere-derived cells express α7 integrin. Graph shows the percentage of α7 integrin expressing cells present in myosphere cultures versus the number of days these cells had remained in culture. Percentage of α7 integrin^+^ cells were determined using flow cytometry. N refers to the number independent myosphere isolations analyzed.

To determine if myospheres contained myogenic cells, myosphere-forming cells were isolated from YFP-Myf5 and YFP-MyoD mice. These mice were made available to me by Dr. Emanuela Gussoni and were obtained by crossing B6.129X1-Gt(ROSA)26Sor mice with Myf5^*Cre*^ or MyoD^*iCre*^ [30] mice. In the ROSA-YFP crossed Myf5^*Cre*^ mice, all cells that have ever expressed the myogenic regulatory factor, Myf5, will express YFP, and in the ROSA-YFP crossed MyoD^*iCre*^ mice, all cells that have ever expressed myogenic regulatory factor, MyoD, will express YFP. Fluorescence microscopy was used to monitor the number of YFP^+^ versus YFP^-^ cells over time, 2 independent isolations were monitored from the YFP-Myf5^*Cre*^ mice and 5 independent isolations from the YFP-MyoD^*iCre*^ mice. Cell counts were taken at the time of the initial cell isolation, during the first 6 days after isolation (before the initial myospheres are formed), and at intermittent time points when the spheres were dissociated into single cell suspensions for passaging. These data as well as examples of myospheres that contain Myf5 and MyoD expressing cells are shown in Figs. 1 and 2, respectively. I found that within 2 hrs of isolation, 8.2 ± 3.6% of myosphere-forming cells expressed Myf5 (Fig. 1a) and 5.8 ± 1.2% expressed MyoD (Fig. 2a). The percentage of cells expressing Myf5 peaked approximately 6 days after isolation (27.2 ± 5.0%) and the percentage of MyoD expressing cells peaked at approximately 3–10 days after isolation (49.6 ± 6.3%). After reaching their peak, the proportion of cells expressing these markers gradually decreased over time (1–2 months after isolation, see Figs. 1 and 2). In addition to expressing Myf5 and MyoD, the presence of myogenic cells was also indicated by the expression of α7 integrin [32,33]. The percentage of cells expressing α7 integrin in myosphere cultures was determined over time using flow cytometry; these results are shown in Fig. 3. I found that within 1 to 3 days of the initial isolation a majority of the cells present expressed α7 integrin (> 60%), however similar to the expression of Myf5 and MyoD, the percentage of cells expressing α7 integrin decreased gradually over time reaching a nadir of < 2% after 36 days in culture. The expression of Myf5, MyoD, and α7 integrin by cells in actively growing myosphere cultures strongly indicates that myogenic cells are present within myospheres.

### Myospheres are composed of two cell populations

**Fig 4:**
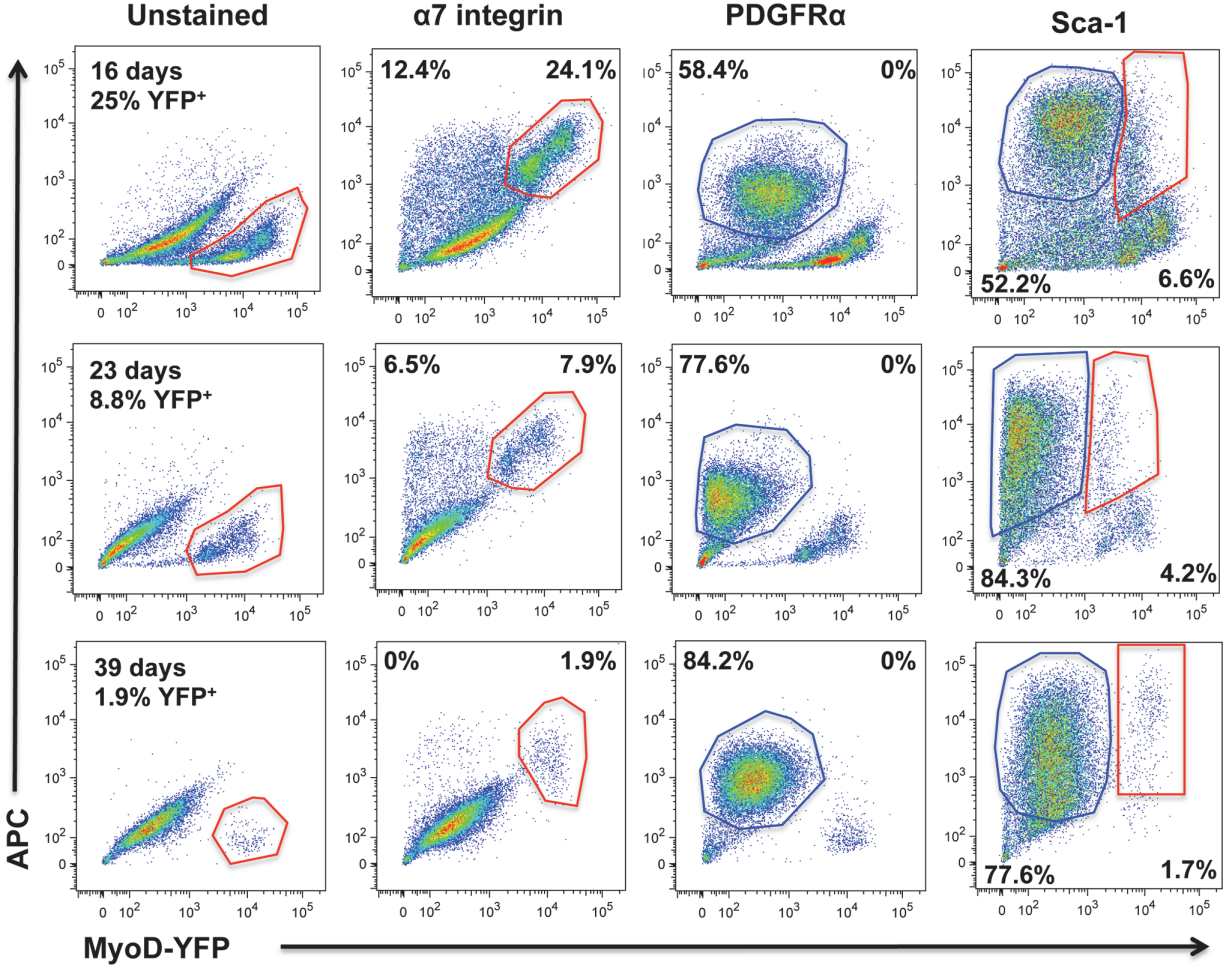
Flow cytometry profiles of myosphere-derived cells isolated from YFP-MyoD mice. Shown are flow cytometry profiles of myosphere-derived cells isolated from YFP-MyoD mice that had remained in culture for 16 (upper panels), 23 (middle panels), and 39 days (lower panels). Shown are the flow cytometry profiles for unstained cells and for cells incubated with APC antibodies against α7-integrin, PDGFRα, and Sca-1. Y-axis shows APC expression and the x-axis YFP expression (MyoD).

Data shown in Figs. 1–3 not only indicate the presence of myogenic cells within myospheres, but also the presence of a second cell type that is non-myogenic. To confirm the myogenic nature of the YFP^+^ myosphere-derived cells isolated from YFP-MyoD mice, as well as to examine the second non-myogenic population of cells within myospheres, I analyzed YFP^+^ and YFP^-^ myosphere-derived cells isolated from YFP-MyoD mice by flow cytometry for the expression of α7 integrin, PDGFRα, and Sca-1. To avoid overlap with the YFP signal, the antibodies used were directly conjugated to APC. These data are shown in Table 1 and Fig. 4. Myosphere-derived cells were analyzed at various time points (between 16 to 39 days after isolation, see Fig. 4) and the percentage of cells expressing α7 integrin, PDGFRα, and Sca-1 within the YFP^+^ and YFP^-^ populations was determined (see Table 1). In accordance with the above data (Figs. 1–3), I found that younger myosphere cultures contained a higher percentage of YFP^+^ cells (25% at 16 days in culture) than older myosphere cultures (< 2% after 39 days in culture). Additionally, in examining the YFP^+^ and YFP^-^ populations, I found that YFP^+^ cells expressed α7 integrin (89.8–100%) but did not express PDGFRα (0%), whereas a majority of the YFP^-^ cells expressed PDGFRα (77.9–85.8%). These data, which indicate that myospheres are composed of two distinct cell populations, are shown in Table 1 and the corresponding flow cytometry profiles for myospheres cultured for 16, 23, and 39 days are shown in Fig. 4.

**Table 1:** Myospheres are composed of two cell types.

Days in Culture	% YFP^+^ Cells	% YFP^+^ cells expressing			% YFP^-^ cells expressing		
		α7 integrin	PDGFRα	Sca-1	α7 integrin	PDGFRα	Sca-1
16*	25.0	96.4	0	26.4	16.5	77.9	69.6
18	17.2	94.8	0	45.9	8.9	82.8	91.9
23*	8.8	89.8	0	47.7	7.1	85.1	92.4
30	6.4	94.8	0	46.7	4.0	84.7	92.1
39^#^	1.8	ND	0	74.1	ND	80.7	98.1
39*	1.9	100	0	89.5	0	85.8	79.1

Table shows diffences in the percent of myosphere-derived cells expressing α7 integrin, PDGFRα, and Sca-1 in YFP^+^ and YFP^-^ cells isolated from YFP-MyoD mice. For each sample 20,000–30,000 cells were analyzed.

* FACS profiles for these cells are shown in Fig. 4.

^#^ Due to a limited number of cells, only 5,300–6,800 cells were analyzed for these samples.

In examining the YFP^-^ cells for the expression of α7 integrin, I also found that younger myospheres contained an YFP^-^ α7 integrin^+^ population of cells (16.5% at 16 days) that was gradually lost over time (0% by 39 days). These data (shown in Table 1 and Fig. 4) along with my previous data obtained from the Myf5 and MyoD lineage-tracing mice (shown in Figs. 1 and 2), indicate that early myosphere cultures contain multiple myogenic cell populations that are at different stages of differentiation, including some that express Myf5 and α7 integrin, but are not yet expressing MyoD.

Additionally, because our previous data showed that Sca-1 is involved in the maintenance of myospheres [25], I analyzed the YFP^+^ and YFP^-^ cell fractions for the expression of Sca-1 (shown in Table 1 and Fig. 4). Interestingly, I found that 26.4% of the myogenic myosphere-derived cells were positive for Sca-1 after being cultured for 16 days, and that the percentage of Sca-1^+^ YFP^+^ cells gradually increased over time (89.5% after 39 days in culture).

**Fig 5:**
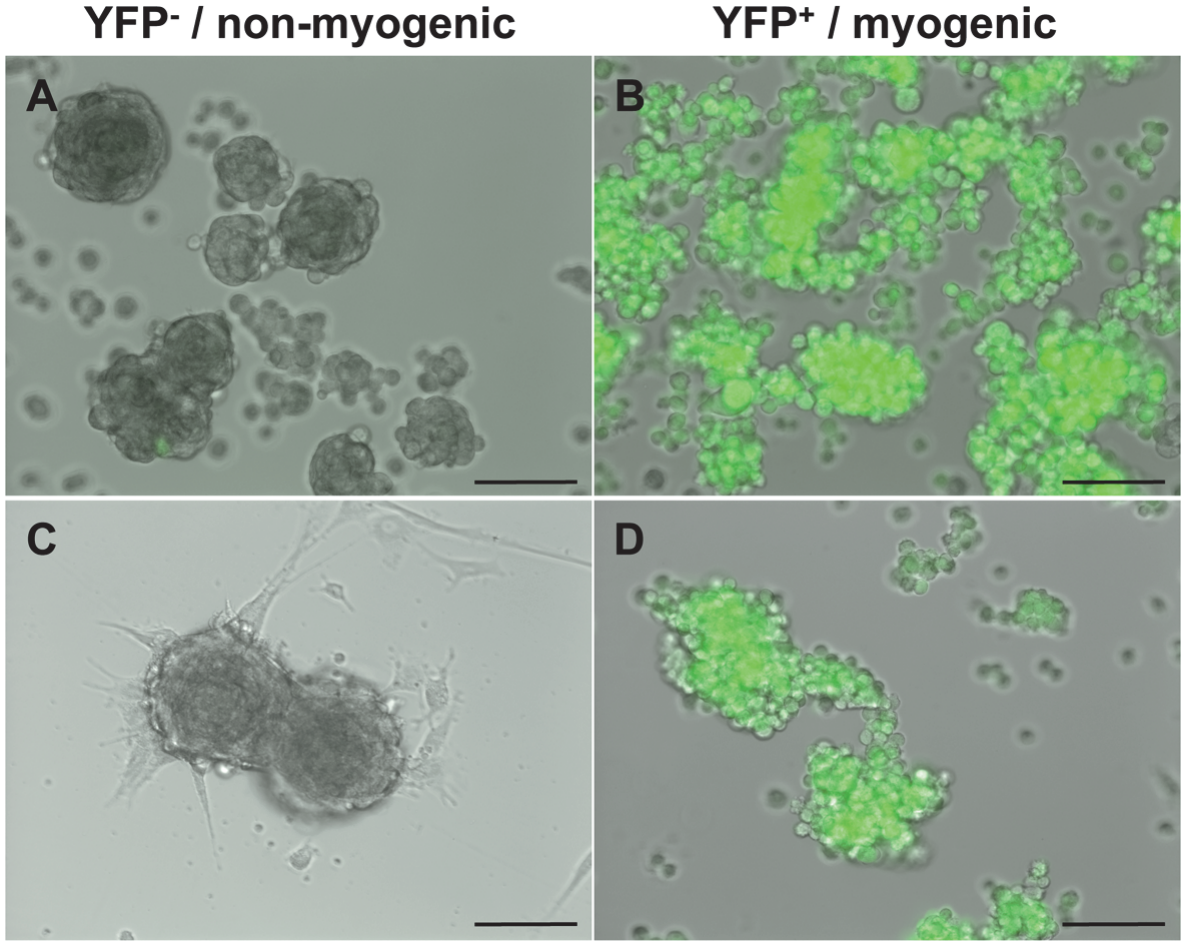
YFP sorted myosphere-derived cells isolated from YFP-MyoD mice. (A-D) Representative pictures showing the growth of YFP^-^ and YFP^+^ sorted myosphere-derived cells isolated from YFP-MyoD mice. Shown are myosphere cultures sorted at (A+B) 18 and (C+D) 39 days after isolation. Upper panels show cells 13 days after sorting and lower panels 17 days after sorting. Scale bar represents 100μm.

Next, to determine if the two cell populations found within myospheres could be cultured independently, myosphere-derived cells isolated from YFP-MyoD mice were sorted into YFP^+^ and YFP^-^ cell populations, plated at equal densities and then formation of new myospheres monitored by fluorescence microscopy over time (see Fig. 5). For this study, two time points were selected, one occurring after the formation of primary myospheres (18 days after isolation, Fig. 5a and 5b) and the second after the formation of secondary myospheres (39 days after isolation, Fig. 5c and 5d). I found that cells that were sorted for the YFP^+^ and YFP^-^ populations could be maintained in culture for about 3–4 weeks and that neither cell population expanded well after sorting. Additionally, upon monitoring the sorted YFP^+^ and YFP^-^ cell populations over time I found that these populations exhibited two distinct behaviors, the myogenic cell population (YFP^+^) remained mostly non-adherent and formed large loosely packed aggregates of floating cells (Fig. 5b and 5d) whereas the mesenchymal cell population (YFP^-^) formed tightly packed spheres that tended to adhere to the floor of the culture dish (Fig. 5a and 5c).

**Fig 6:**
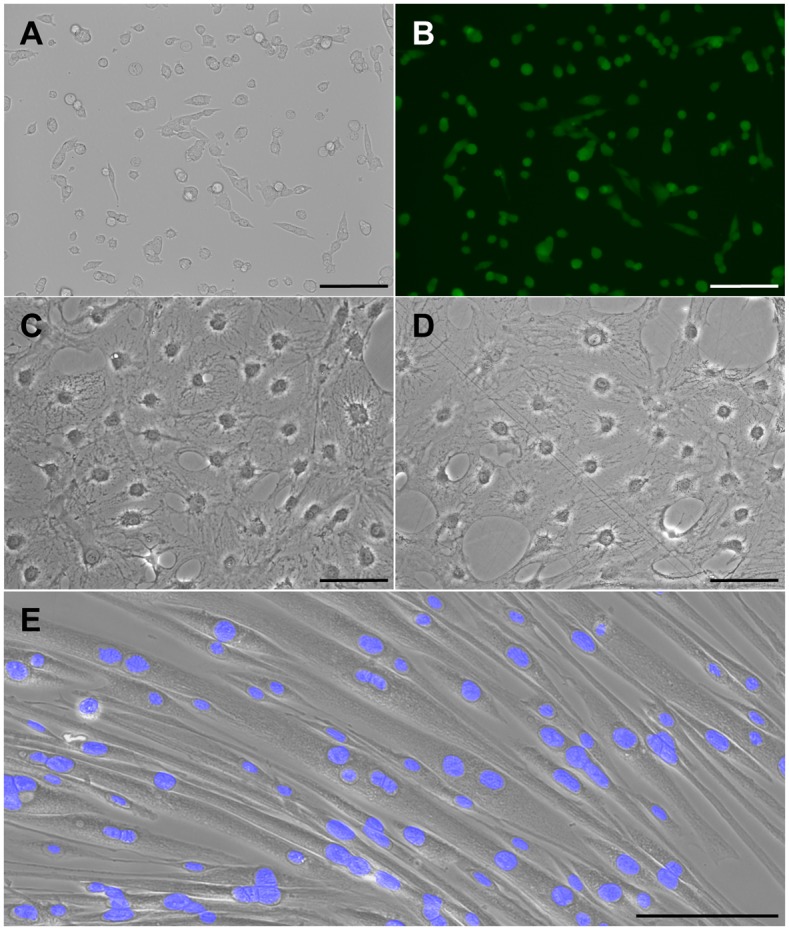
Adherently cultured myosphere-derived cells. Representative pictures showing adherently cultured myosphere-derived cells that had been sorted for the (A–B) MyoD-YFP^+^, (C) α7 integrin^-^, and (D) PDGFRα^+^ populations. (E) Representative picture showing the formation of myotubes by adherently cultured MyoD-YFP^+^ myosphere-derived cells. Nuclear staining by DAPI, shown in blue, is used to show the formation of multinucleated myotubes. Scale bar represents 100μm.

Additionally, when sorted myosphere-derived cells were cultured adherently in myoblast media (F10 media containing 20% FCS), I found that the cells associated with the myogenic fractions (YPF^+^ / α7 integrin^+^ / PDGFRα^-^) maintained a smaller myoblast-like morphology and could be easily passaged in culture (Fig. 6a and 6b), whereas the cells associated with the mesenchymal fraction (YPF^-^ / α7 integrin^-^ / PDGFRα^+^) were larger cells that maintained a flatten mesenchymal-like morphology (Fig. 6c and 6d); these cells were difficult to maintain in culture (< 2 passages) even when I tried culturing them using several different media and serum combinations. The differences in morphology between the adherently cultured myogenic and mesenchymal-like myosphere cells are shown in Fig. 6. Furthermore, to confirm the myogenicity of the cells isolated from myospheres, adherently cultured YFP-MyoD myosphere-derived cells, which had been sorted for the myogenic (YFP^+^) and mesenchymal (YFP^-^) cell populations, were plated on ECL coated wells and monitored for the formation of myotubes (see Fig. 6e). I found that the adherently cultured YFP^+^ myosphere-derived cells were able to form multinucleated myotubes, whereas the adherently cultured YFP^-^ cells did not readily form myotubes.

### Myogenic myosphere-derived cells retain CellTrace dye

**Fig 7:**
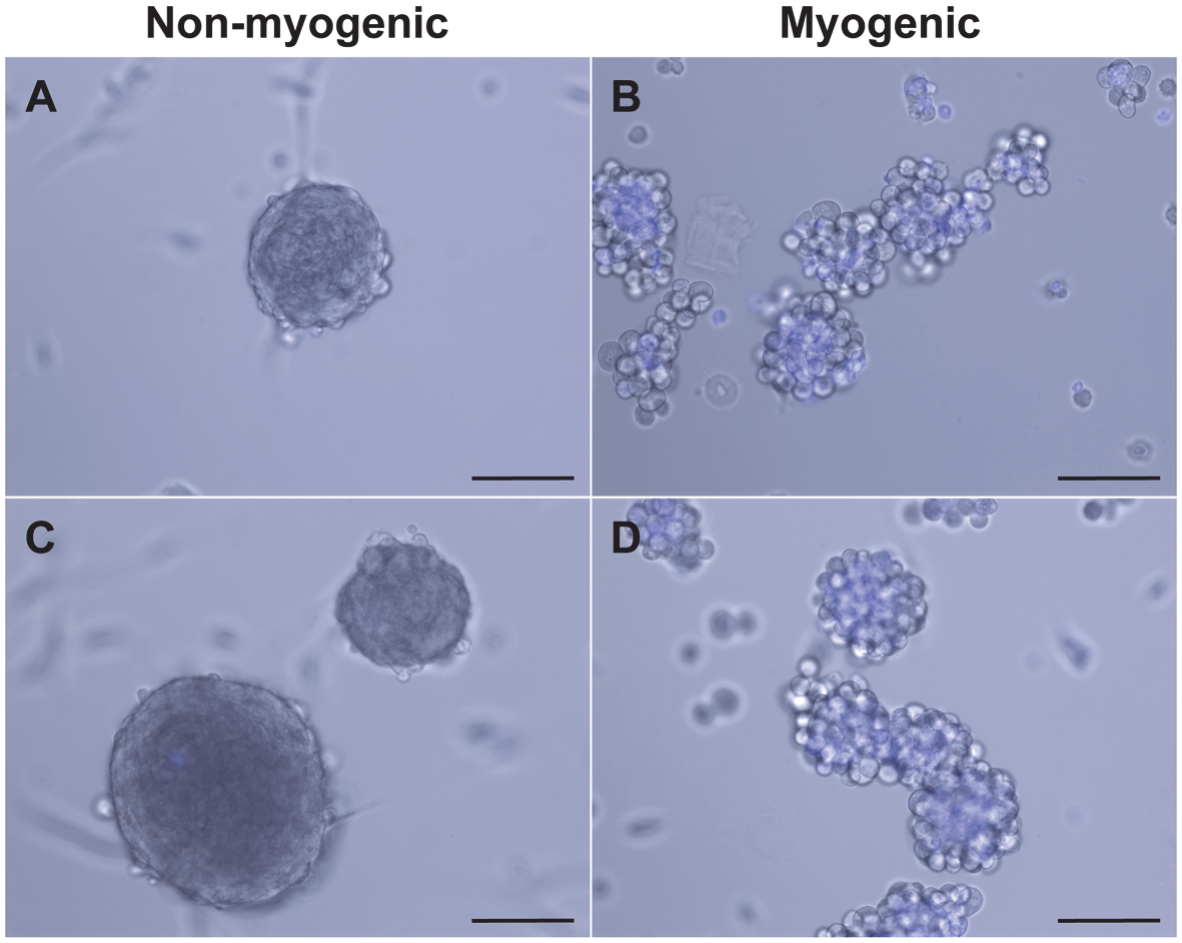
CellTrace labeled myosphere-derived cells sorted into non-myogenic and myogenic cell populations. Representative pictures showing the growth of CellTrace labeled myosphere cells sorted into (A) α7 integrin^-^, (B) α7 integrin^+^, (C) PDGFRα^+^, and (D) PDGFRα^-^ cell populations. Cells sorted for (A) α7 integrin^-^ and (C) PDGFRα^+^ correspond to the non-myogenic mesenchymal myosphere-derived cell population and cells sorted for (B) α7 integrin^+^ and (D) PDGFRα^-^ correspond with the myogenic myosphere-derived cell population. Myosphere cultures shown were sorted 9 days after isolation; pictures were taken 12 days after sorting. Scale bar represents 100μm.

To determine if the loss of myogenic cells within myospheres could be due to these cells dividing at a different rate than the mesenchymal myosphere cells, I used CellTrace Violet to label myosphere cells isolated from *wt* mice. CellTrace works by diffusing into the cell where it is cleaved by intracellular esterases to yield a fluorescent compound, which binds covalently to intracellular amines, resulting in cell staining that is stable over time but becomes more dilute when the cell divides. After labeling with CellTrace, cells were sorted for CellTrace positive cells that displayed either myogenic (α7 integrin^+^ / PDGFRα^-^) or mesenchymal (α7 integrin^-^ / PDGFRα^+^) markers, these cells were then plated at equal density and then their growth monitored over time using fluorescence microscopy. These results are shown in Fig. 7. I found that within a few days of sorting, cells associated with the mesenchymal cell population (those that were α7 integrin^-^ or PDGFRα^+^) had already lost the CellTrace dye (shown in Fig. 7a and 7c, respectively), whereas the myogenic cell population (those that were α7 integrin^+^ or PDGFRα^-^) retained the CellTrace dye for >20 days (shown in Fig. 7b and 7d, respectively), indicating that the mesenchymal population of cells were dividing at a much faster rate than their myogenic counterparts. Additionally, similar to what was seen with the YFP-MyoD sorted cells (shown in Fig. 5), the sorted myogenic populations remained non-adherent and formed large loosely packed aggregates of floating cells (Fig. 7b and 7d) whereas the mesenchymal populations formed tightly packed spheres that tended to adhere to the floor of the culture dish (Fig. 7a and 7c).

**Fig 8:**
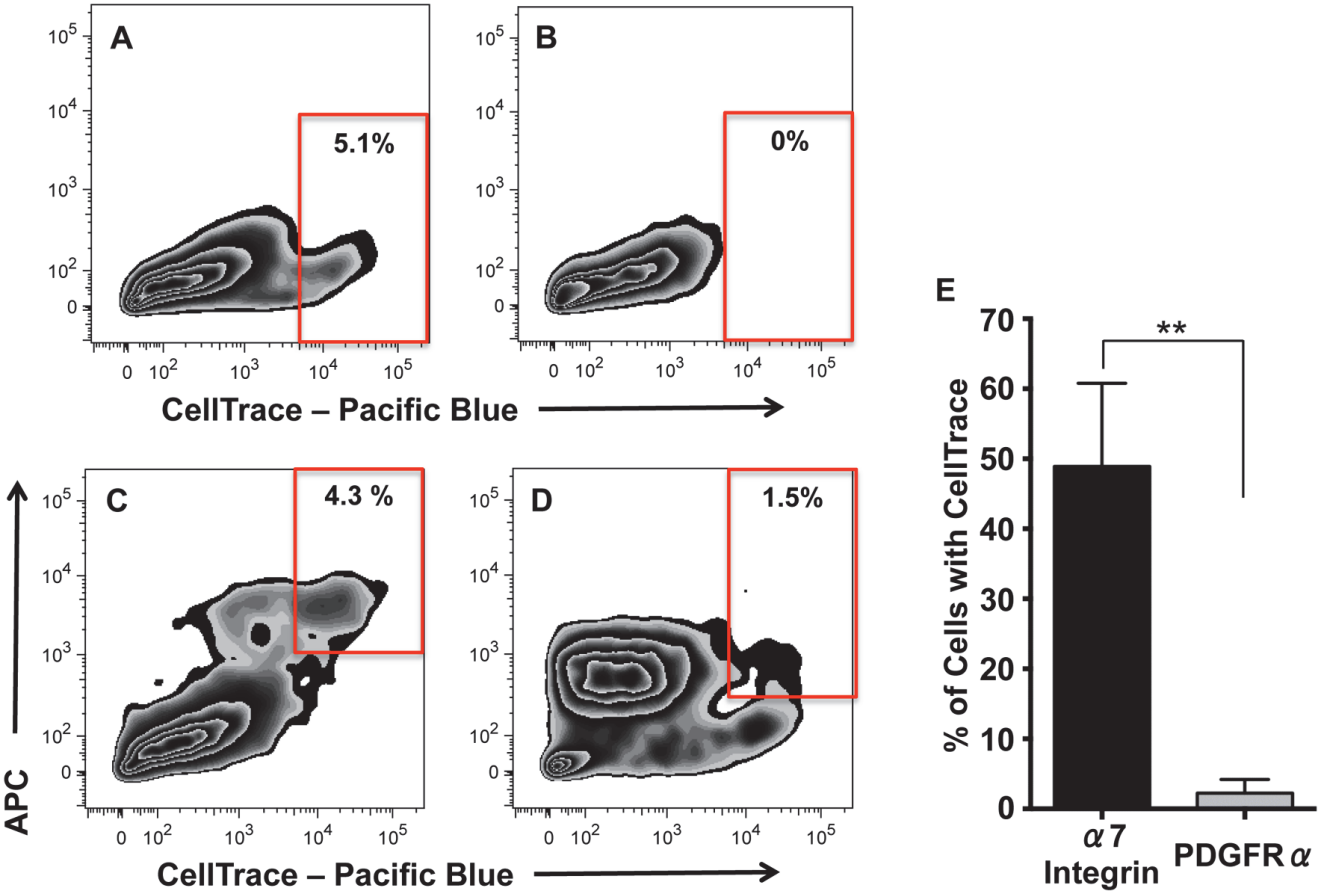
Flow cytometry profiles of CellTrace labeled myosphere-derived cells. (A–B) Representative flow cytometry profiles of (A) younger and (B) older CellTrace labeled myosphere-derived cells. Myosphere-derived cells represented in (A) had remained in culture for 16–24 days and those in (B) for >36 days. (C–D) Representative flow cytometry profiles showing the expression of (C) α7 integrin and (D) PDGFRα in 16 day old CellTrace labeled myosphere-derived cells. (E) Graph showing the percentage of myosphere-derived cells that retain CellTrace label while expressing α7 integrin or PDGFRα. N = 4 independent isolations for the data presented in (A–E). **p = 0.0037 by unpaired Student’s t test.

To eliminate the possibility that the process of sorting may have influenced the growth of one or both of the cell populations, myospheres were dissociated at different time points, labeled with CellTrace, cultured for 7–10 days without sorting, and then analyzed using flow cytometry. These results are shown in Fig. 8. Fluorescence microscopy was used to confirm that all the cells had initially received the CellTrace label and to monitor the retention of the CellTrace label over time as the cells formed myospheres. Initial observations indicated that younger myospheres retained more CellTrace label than older myospheres. These observations were confirmed using flow cytometry to measure the degree of CellTrace remaining in myosphere cells; retained CellTrace label could be seen in flow cytometry profiles as a tail that extended out to the right (shown in Fig. 8a). Analysis of this tail showed that 5.9 ± 2.4% (N = 4) of myosphere cells, which had remained in culture for 16–24 days, retained high levels of CellTrace, whereas there was no tail seen in the CellTrace flow cytometry profiles of myosphere cells that had remained in culture for greater than 36 days (N = 4) (Fig. 8a and 8b, respectively). These data indicated that there is a population of cells dividing at a slower rate in younger myosphere cultures that is lost in the older myosphere cultures. Additionally, to determine if the retention of CellTrace label by younger myospheres was associated with the myogenic, mesenchymal, or with both of these myosphere cell populations, flow cytometry was used to analyze CellTrace labeled myosphere cells for the expression of α7 integrin and PDGFRα (Fig. 8c and 8d, respectively). The retention of CellTrace by younger myospheres was found to be associated with the myogenic population of myosphere-derived cells (α7 integrin^+^ cells, Fig. 8c), where 48.9 ± 2.2% (N = 4) of the α7 integrin^+^ cells also retained high levels of CellTrace, versus only 2.2 ± 1.0% (N = 4) of the PDGFRα^+^ cells (Fig. 8d), indicating that in younger myosphere cultures the mesenchymal population of cells divide at a significantly faster rate than their myogenic counterparts (Fig. 8e). Additionally, I found that some of the myogenic cells retained greater amounts of the CellTrace dye than others, indicating that within the myogenic population there is a subpopulation of cells that are either not dividing or dividing very slowly.

### Pax7^+^ cells are present within myospheres

**Fig 9:**
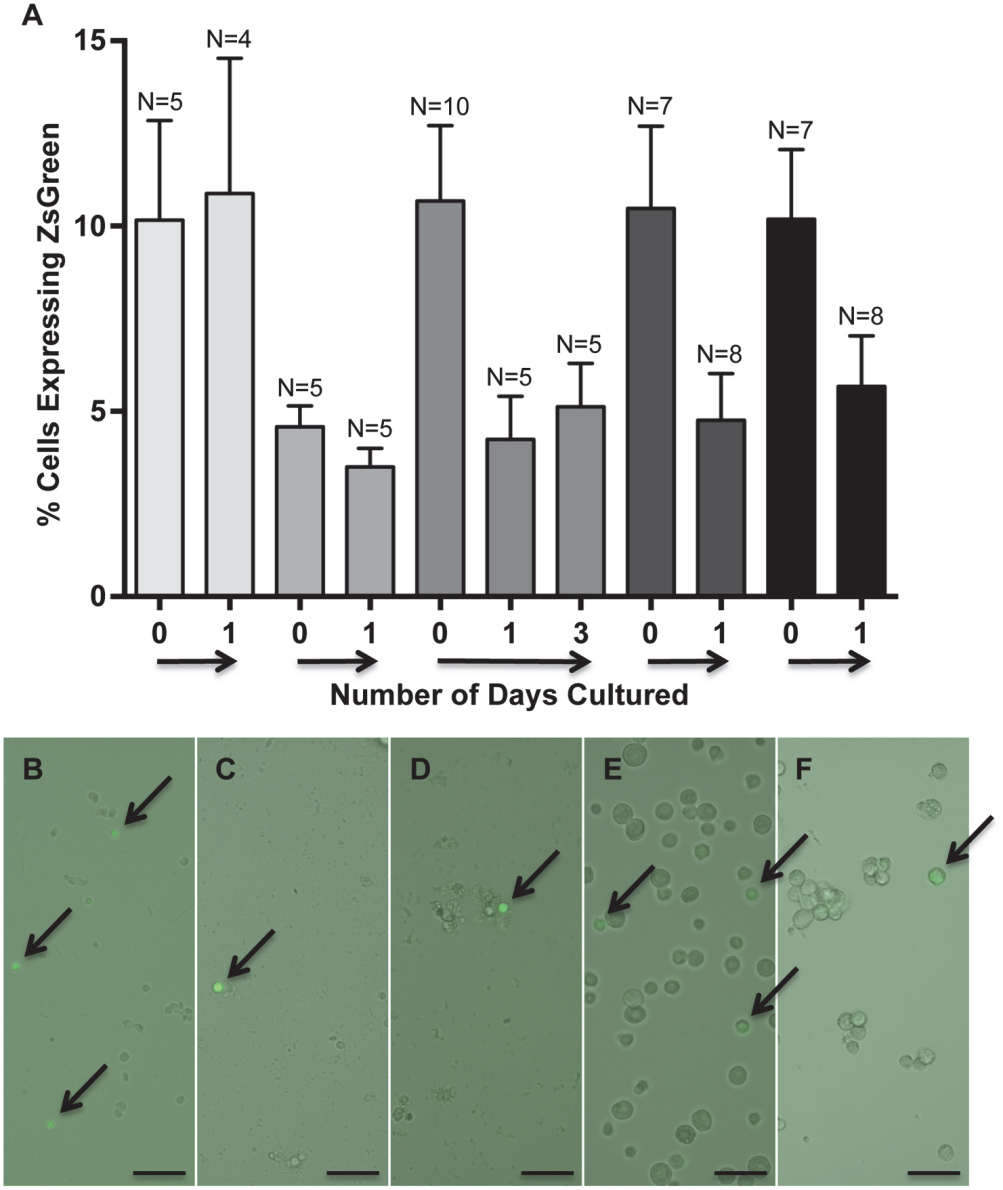
Myosphere-derived cells express Pax7. (A) Graph showing the percentage of myosphere-derived cells isolated from ZsGreen-Pax7 mice that express ZsGreen versus the number of days these cells had remained in culture. Shown are five independent myosphere isolations. N refers to the number of random fields counted. (B-F) Representative pictures showing the growth of myospheres and the expression of ZsGreen in myosphere cultures isolated from ZsGreen-Pax7 mice. Arrows indicate ZsGreen^+^ cells. Shown are pictures from (B) 0, (C) 1, (D) 3, (E) 8, and (F) 15 days after isolation. Cells shown in (E) had been sorted for the expression of α7 integrin. Scale bar represents 50μm.

**Fig 10:**
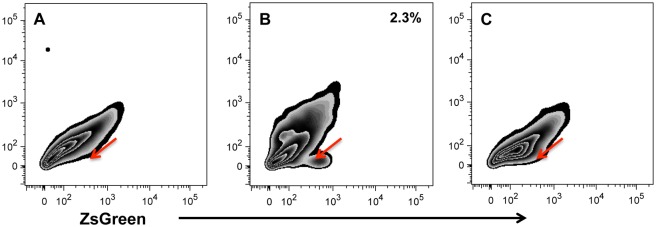
Flow cytometry profiles of myosphere-derived cells isolated from ZsGreen-Pax7 mice. Shown are representative flow cytometry profiles for myosphere-derived cells that were isolated from (A) wild type and (B-C) ZsGreen-Pax7 mice. Myosphere-derived cells represented in (B) have remained in culture for 1 day and those in (C) for 9 days. Red arrows indicate location of the ZsGreen^+^ cell population. N = 6 independent isolations were analyzed for the data presented in (B), and N = 6 for the data presented in (C). Five of the six isolations in (B) were analyzed for 100,000 counts.

To determine if the slowly dividing myogenic cells within myospheres could be satellite cells, myosphere-forming cells were isolated from ZsGreen Pax7 mice [31]. In these mice, the expression of ZsGreen is driven by the Pax7 promoter, so cells expressing Pax7 will also express ZsGreen, however unlike the YFP-MyoD^*iCre*^ cells, when the promoter is no longer active, the ZsGreen expression fades and is quickly lost. As done previously, fluorescence microscopy was used to monitor myosphere isolations from ZsGreen-Pax7 mice. These results are shown in Fig. 9. I found that immediately after isolation 9.2 ± 1.2% of myosphere cells expressed Pax7 (N = 5), which decreased to 6.9 ± 1.2% one day after isolation (N = 7), and by 3 days after isolation it became difficult to determine the percentage of ZsGreen^+^ cells because the number of cells present increased, resulting in a higher level of background fluorescence, at the same time that the intensity of ZsGreen fluorescence began to fade. Nevertheless, although the intensity of ZsGreen had started to fade just a few days after isolating myosphere cells, ZsGreen^+^ cells could still be seen in myosphere cultures for at least 15 days after isolation (see Fig. 9f). The percentage of cells expressing Pax7 was also determined by flow cytometry (see Fig. 10). Similar to the microscopy results, I found that intensity of ZsGreen expression was greatest within a day or two of the initial isolation (Fig. 10b) and then faded over time (Fig. 10c); this can be seen in the flow cytometry profiles as a shift to the right of the main population of cells (1 day after isolation, shown in Fig. 10b), the shift later becomes less pronounced and is seen as a bump that extends to the right (8–18 days after isolation, shown in Fig. 10C), and by day 24 the flow cytometry profiles became indistinguishable from those obtained from myosphere-derived cells isolated from wild type mice. I found that 2.3 ± 0.2% of myosphere-derived cells strongly expressed ZsGreen one day after isolation (N = 6, see Fig. 10b) and that although there was clearly a shift in the flow cytometry profiles of cells analyzed at 8–18 days after isolation, it was difficult to determine the percentage of cells expressing ZsGreen because this cell population had begun to merge into the main cell population (N = 6, see Fig. 10C). These data indicate that there are Pax7 positive cells initially present in myosphere cultures, however like Myf5, MyoD, and α7 integrin expressing cells, they are lost over time.

## Discussion

The possible origin of myospheres has been controversial. Previously, to avoid altering the properties of the non-adherent myosphere-derived cells, we used PCR to determine if myospheres expressed myogenic markers. The results of that study, which was done using well established myospheres that had remained in culture for > 2 months, indicated that myospheres themselves did not express myogenic markers, but that upon differentiation their progeny did express these markers [23]. Other researchers, who used immunofluorescence to detect the expression of MyoD and Pax7, reported that myospheres do contain myogenic cells [34,35] however like us, they also reported these cells were able to differentiate into adipogenic and osteogenic cells indicating myosphere cells had a mesenchymal nature [23,34,35]. In this study, to clearly determine if myospheres are composed of myogenic cells without altering the structure of the myospheres, I isolated myosphere-forming cells from lineage-tracing mice and then monitored their growth over time. Using these mice I found that early myosphere cultures do in fact contain myogenic cells that express Myf5, MyoD, and Pax7. Additionally, although cells expressing these markers peaked at different times, Pax7 cells within 2 days of isolation, Myf5 at around 6 days, and MyoD in a range between 3–10 days after isolation, indicating that some of these cells had become activated and expanded, I also found that there was an overall loss in the number myogenic cells (α7 integrin^+^) over time. This gradual loss of myogenic cells lead me to wonder if these cells are dividing at a slower rate than their non-myogenic counterparts. To determine if differences in cell proliferation played a role in the loss of myogenic cells, I used CellTrace to label the slower dividing cells. I found that the cells, which retained the greatest amount of CellTrace label, corresponded with the myogenic population of cells (those that were α7 integrin^+^ / PDGFRα^-^), indicating that the loss of the myogenic population of cells may be due to these cells dividing slower rate than their non-myogenic counterparts. Additionally, I also found that within the myogenic population of cells, some cells retained a greater amount of CellTrace label than others, indicating that within the myogenic population of cells there is a small subpopulation that is either not dividing or dividing very slowly. The data presented here, which shows that Pax7 expressing cells are present in myosphere cultures, along with our previous data showing that myosphere-derived cells can be used to regenerate injured muscle [23], indicate that myospheres not only contain myogenic cells but that within the myogenic cell population there is a small subpopulation of Pax7^+^ satellite cells.

Additionally, because our previous studies indicated that the expression of Sca-1 was important for the formation and maintenance of myospheres [25], here I analyzed the myosphere-derived myogenic and mesenchymal cell populations for the expression of Sca-1. Interestingly, I found that there was a population of myosphere-derived myogenic cells that expressed Sca-1, and that over time the percentage of myogenic cells expressing Sca-1 increased. This increase appeared to correlate with a decrease in the percentage of myogenic cells present within the myosphere cultures. A possible explanation for the expression of Sca-1 by this myogenic cell population, is that the expression of Sca-1 may be due to the environmental conditions generated by this particular culturing system, that is, the expression of Sca-1 by these cells may be a cell survival mechanism related to the non-adherent nature of the myosphere culture [25,36].

By using the lineage-tracing mice to mark the myogenic cells, I found that myospheres also contained a population of cells that were non-myogenic. This second population of cells was found to express PDGFRα and Sca-1, both of which have been associated with muscle-derived fibro/adipogenic mesenchymal cells [27,28]. To determine if these two myosphere-derived cell populations could be cultured independently, I monitored the growth of myosphere-derived cells that had been sorted for markers corresponding with either the myogenic (MyoD-YFP^+^, α7 integrin^+^, or PDGFRα^-^) or the mesenchymal (MyoD-YFP^-^, α7 integrin^-^, or PDGFRα^+^) populations of cells. After sorting I found that the sorted cells exhibited two distinct behaviors in culture, cells that were associated with the myogenic populations formed lose clusters that remained non-adherent, whereas cells that were associated with the mesenchymal populations formed tight spheres that tended to adhere to the bottom of the plate. Essentially I found that there was a change in both the growth and morphology of myospheres that were composed of only myogenic or mesenchymal cells, indicating that perhaps both cell types are involved in maintaining this unique system in culture. Interestingly, *in vivo* studies also indicate that there is an interaction occurring between these cells and that this interaction may play an important role in the maintenance and survival of satellite cells: one study showed that the ablation of Tcf4 fibroblasts altered satellite cell dynamics resulting in their premature activation and depletion of the satellite cell pool [16], a second linked the survival of Sca-1^+^ muscle mesenchymal cells after injury to the presence of α7 integrin positive cells [37], and a third found that the loss of FAP expressing stromal cells, significantly reduced muscle mass [38]. Additionally, the importance of non-satellite / mesenchymal type cells to muscle regeneration was also indicated in two recent reviews in which these cells are seen to play a regulatory-paracrine type role that influences the proliferation and differentiation of satellite cells [39,40]. The commonality in these reports is that they all point to an interplay that occurs within muscle between myogenic and mesenchymal-like cells, as well as strongly indicate that this interplay is needed for the proper maintenance of muscle. Interestingly, here I present data demonstrating that myospheres are composed of two cell types, one myogenic and the other mesenchymal, and although future studies are needed to fully understand this unique co-culturing system, I believe that this system could be used to more closely mimic the *in vivo* satellite cell environment, which could then enable potential muscle donor cells (such as primitive satellite cells) to remain in culture for longer periods of time.
